# Deviation-support based fuzzy ensemble of multi-modal deep learning classifiers for breast cancer prognosis prediction

**DOI:** 10.1038/s41598-023-47543-5

**Published:** 2023-12-03

**Authors:** Nikhilanand Arya, Sriparna Saha

**Affiliations:** Department of Computer Science & Engineering, Indian Institute of Technology Patna, Bihar, 801106 India

**Keywords:** Breast cancer, Machine learning

## Abstract

Breast cancer is the fifth leading cause of death in females worldwide. Early detection and treatment are crucial for improving health outcomes and preventing more serious conditions. Analyzing diverse information from multiple sources without errors, particularly with the growing burden of cancer cases, is a daunting task for humans. In this study, our main objective is to improve the accuracy of breast cancer survival prediction using a novel ensemble approach. It is novel due to the consideration of deviation (closeness between predicted classes and actual classes) and support (sparsity between predicted classes and actual classes) of the predicted class with respect to the actual class, a feature lacking in traditional ensembles. The ensemble uses fuzzy integrals on support and deviation scores from base classifiers to calculate aggregated scores while considering how confident or uncertain each classifier is. The proposed ensemble mechanism has been evaluated on a multi-modal breast cancer dataset of breast tumors collected from participants in the METABRIC trial. The proposed architecture proves its efficiency by achieving the accuracy, sensitivity, F_1_-score, and balanced accuracy of 82.88%, 58.64%, 62.94%, and 74.75% respectively. The obtained results are superior to the performance of individual classifiers and existing ensemble approaches.

## Introduction

Breast cancer, the most commonly diagnosed cancer globally, has exceeded lung cancer in diagnoses, with 2,261,419 cases reported in women in 2020 according to the https://www.cancer.net/cancer-types/breast-cancer/statistics survey. In the same year, 684,996 deaths happened, and this disease became the fifth leading cause of death in females. The majority of the deaths are caused by invasive breast cancers, where cancerous cells are not limited to the breast region and spread across other body parts of the patient. For the year 2023, the survey estimated 297,790 women in the United States will be diagnosed with invasive breast cancer and 43,170 deaths will occur. The following subsection details the significance and challenges associated, potential of deep learning and our contribution towards the breast cancer survival prognosis.

### Significance and challenges of breast cancer survival prognosis

Breast cancer patients are often confronted with pressing questions such as “What is the extent of my cancer?” and “What are my chances of survival?” These inquiries are addressed through a process known as prognosis, which provides insights into the disease’s trajectory, particularly in its early stages. Discussions about prognosis are crucial for patients to gain a comprehensive understanding of their disease’s likely progression. Favorable prognoses can alleviate the anxiety accompanying a cancer diagnosis, while unfavorable prognoses might encourage patients to engage in end-of-life conversations, establish advance directives, and address related concerns. Moreover, a patient’s prognosis plays a pivotal role in shaping treatment decisions. Those with promising prognoses might opt for aggressive treatments in pursuit of remission, while those with less promising prognoses could choose palliative treatments to enhance their quality of life and mitigate side effects.

Survival analysis is the most essential study for any oncologist to identify the severity and chances of recovery of a cancer patient. Oncologists utilize different sources like clinical details, genetics details, and histopathological images for survival analysis with the standard survival rate of a 5-year cutoff. Based on the survival analysis, they plan the treatment of the patients in an efficient way where minimal toxic side effects and financial burden^1^ with zero overtreatments are ensured. The correct survival analysis requires an expert oncologist with years of experience, as breast cancer varies significantly from person to person resulting in random clinical outcomes^2^. Complexity and heterogeneity present in the breast cancer clinical and genomic data make the survival prognosis an erroneous task.

### Potentials of deep learning

Complex and high-dimensional data is the bottleneck for any human mind, while it is the treasure for deep learning models. Moreover, multi-modal data generates a massive feature space with complex data patterns if fused together. The extraction of handcrafted features from multi-modal data becomes a tedious job due to intricate data patterns, and there remain higher chances of missing some informative features. Deep learning architectures overcome this limitation with their characteristics of self-learning complex data patterns and hidden relationships without the help of feature engineering. This establishes the usefulness of deep learning in the breast cancer survival analysis task. There are plenty of deep learning architectures possible, and they can identify different underlying relationships between the input data and the class labels, resulting in different classification abilities. The ensemble learning is a mechanism where prediction scores of two or more classifiers aggregate to generate the final decision for any classification task. It has proven its way for more robust classification architectures and reliable results. However, the conventional ensembling methods do not consider the fact that different classifiers might have different distributions of prediction scores and treat all of them as having the same importance.

### Our contribution

Considering this limitation of the existing ensemble approach, we propose a Deviation-Support based Fuzzy Ensemble (*DeSuFEn*) method. The proposed ensemble is novel due to the incorporation of deviation and support terms, which signifies the confidence of base classifiers in the ensemble mechanism. These terms identify the margin between the actual and expected prediction probabilities. The deviation term indicates the gap between predicted probability and actual probability and acts as the penalty. The support term is the measure of closeness between the predicted and actual probabilities and works as the reward. These terms are defined with the help of two non-linear functions of different concavities, where one follows *exponential*, and the other follows *tanh* characteristics. The higher the value of the prediction probability, the more support and less deviation from the actual class is observed. Further, the ensemble method parallelly integrates the values of deviations or supports over the given set of base classifiers under the Choquet fuzzy integral. The fuzzy integral blends deviations or supports in a way that considers how confident or uncertain each classifier is. It combines these diverse inputs to make a more informed decision, acknowledging that some sources might be less sure than others. For this task, it calculates the memberships of all possible combinations of classifiers and then provides the aggregated scores. These aggregated support and deviation scores are further utilized for the final prediction. The deviation and support functions are selected to keep the strong influence of deviation in the final prediction, where the final prediction is decided by the agreement of both aggregated support and deviation scores toward a particular class. In case of a clash between these two, our strategy prefers the deviation score and its relevant class as the final prediction. The goal of the ensembling strategy is to minimize the deviation and maximize the support values. The ensemble approach in this work uses four base classifiers, namely, SiGaAtCNN STACKED RF, SiGaAtCNN+Input STACKED RF, BiAttention, and BiAttention STACKED RF from the work of Arya et al.^3^. All these base classifiers are the multi-modal deep learning architectures trained on multi-modal data (consisting of clinical, gene expression, and copy number variation details of breast cancer patients) from the METABRIC^4^ trail. The task of these base classifiers is to classify breast cancer patients as short-term and long-term survivors based on the standard 5-year survival cutoff using multi-modal data. The fuzzy ensemble techniques utilized by Pranab et. al.^5, 6^ motivated us to develop the proposed *DeSuFEn*. It handles the limitations of already existing ensemble methods and aggregates the prediction scores of base classifiers in a fuzzy manner, where the inclusion of confidence in prediction is guaranteed. The proposed ensemble is superior in its ensembling strategy and has lower time complexity for multi-modal breast cancer survival prediction. The contributions of the proposed study are as follows:

1. Ensemble learning using state-of-the-art base classifiers SiGaAtCNN STACKED RF, SiGaAtCNN+Input STACKED RF, BiAttention, and BiAttention STACKED RF to enhance the performance of the modal in the presence of high-class imbalance data for the task of breast cancer survival prediction.
2. The ensemble learning uses two non-linear functions as the quantified measure of support and deviation for the prediction scores towards the actual class. These quantified measures generated by four state-of-the-art the base classifiers are ensembled using the Choquet fuzzy integral to get the aggregated support and deviation values. The aggregated support and deviation values determine the final prediction class.
3. The proposal of deviation and support functions and their Choquet fuzzy ensemble is novel and shows its efficacy with the boost in the performance measures.
4. For the empirical validation of the proposed technique, we have used the multi-modal METABRIC^4^ dataset of breast cancer patients and trained our model in a 10-fold cross-validation framework. The results obtained from the experiments show the superiority of our ensembling approach.

## Literature review

The study on breast cancer prognosis and diagnosis using machine learning techniques started in the early 90's. In the starting days, gene expression was the prevalent and only source of information for cancer prognosis. Several researchers mainly focused on identifying signature genes responsible for the occurrence of breast cancer^7, 8^. With the popularity of machine learning algorithms, some studies utilized Support Vector Machine (SVM)^8^ and Random Forest (RF)^9^ in the task of breast cancer prognosis prediction. The machine learning models are suitable for any prediction task only if the data is handcrafted or some feature selection technique has been applied; otherwise, it fails. Hence, Xu et al.^8^ and Nguyen et al.^9^ applied SVM and Bayesian probability based feature ranking and selection in their works, respectively. The advancement of medical data extraction techniques resulted in the availability of various clinical features and histopathological tissue slides. All these data sources evolved as multi-modal data, and research started on multi-modal breast cancer prognosis and diagnosis^3, 10, 11^. In the direction of our classification goal, Sun et al.^10^ proposed MDNNMD as the multi-modal deep neural network for the survival prediction of breast cancer patients. They performed a weighted score fusion of three independent deep neural networks of clinical, gene expression, and copy number variation data, which outperformed some popular machine learning algorithms such as SVM, RF, and logistic regression (LR). Further Arya et al.^3, 11^ showcased the efficiency of convolutional neural networks (CNN) as feature extractors and trained three different CNNs for the above-mentioned three modalities. Their models are stacked architectures where random forest classifiers are stacked upon the CNNs. The random forest is trained to predict survival using the hidden features extracted from CNNs. In the extension, they also explored the availability of additional modalities and proposed *logCosh* variational auto-encoders (VAE)^12^ feature-based breast cancer survival prognosis.

A thorough literature survey resulted in several machine-learning^8, 9, 13^ and deep-learning models^3, 11, 12, 14, 15^ for breast cancer prognosis prediction ranging from uni-modal to multi-modal. Clinical modality-based uni-modal ensembles^16^ have shown enhancements over base classifiers in the breast cancer survival prediction task. However, we did not find any work where multi-modal base classifiers are ensembled. Table 1 provides a summarized view of the research gaps of the above-mentioned approaches in the field of breast cancer prognosis prediction.

**Table 1:** Comparison of numerous approaches applied for breast cancer prognosis prediction.

Approach	Clinical	Gene expression	Copy number alteration	Multi modal	Cross validation	Machine learning	Deep learning	Ensemble learning
Arya and Saha^3^	✓	✓	✓	✓	✓	✓	✓	×
Vijver et al.^7^	×	✓	×	×	×	✓	×	×
Xu et al.^8^	×	✓	×	×	×	✓	×	×
Nguyen et al.^9^	✓	×	×	×	✓	✓	×	×
Sun et al.^10^	✓	✓	✓	✓	✓	×	✓	×
Arya and Saha^11^	✓	✓	✓	✓	✓	✓	✓	×
Arya and Saha^12^	✓	✓	✓	✓	✓	✓	✓	×
Arya and Saha^13^	✓	✓	✓	✓	✓	✓	×	×
Zhang et al.^15^	✓	×	×	×	✓	×	✓	×
Sweetlin et al.^16^	✓	×	×	×	✓	✓	×	✓
Gevaert et al.^17^	✓	✓	×	✓	✓	✓	×	×
Palmal et al.^18^	✓	✓	✓	✓	✓	×	✓	✓

A slight leniency in the filtering criteria and search of ensemble methods in other problem statements resulted in some simple aggregation techniques. Sarwar et al.^19^ used an average probability-based two-level ensemble for screening cervical cancer using Papanicolaou smear image analysis. Pre-trained CNN-based COVID-19 classification^20, 21^ and pathogen classification^22^ are a few instances where a simple averaging ensemble has proven its efficacy. Xue et al.^23^ used a majority voting ensemble of pre-trained CNNs for cervical histopathology image classification. The existing ensemble methods have a major drawback that these are inconsiderate of the confidence in the predictions and give predetermined fixed importance to the base classifiers. Ekbal and Saha^24^ developed a weighted vote-based classifier ensemble to determine the appropriate weights of voting for each class in each classifier using a genetic algorithm for the named entity recognition task. Further, they also explored the simulated annealing multi-objective optimization-based ensemble of classifiers for named entity recognition^25^ and part of speech tagging^26^ tasks. The genetic algorithm and simulated annealing multi-objective optimization techniques raised the concern of high time complexity in identifying the weights of voting for each class in each classifier.

## Dataset

METABRIC^4^ provides the multi-modal data of breast cancer patients in clinical, gene expression, and copy number variation profiles. It has the patient’s survival details embedded inside the clinical modality. For our study, we select 1980 cancer patients from this trial and attach binary labels to them. The label information is fetched from the clinical profiles categorizing patients as long (labeled 0) and short (labeled 1) term survivors based on the standard 5-year survival cutoff. The data shows a class imbalance that is highly biased toward the long-term survivors with 1489 patients. As per the statistics of Cancer.Net, the median age of being diagnosed with this disease is 63 years. The METABRIC data also has 61 years as the median age at diagnosis. Instead of using raw data and applying feature engineering from scratch, we acquired the engineered data from the GitHub repository of Sun et al.^10^. Here, missing values of the genetic profiles are filled with weighted nearest neighbor algorithm^27^. The normalization followed by discretization is performed on the gene expression data to categorically identify the level of expressed genes. These categories include under-expression (− 1), baseline (0), and over-expression (+1) of genes based on the calculated threshold^28^. The copy number variation features are already available in discrete forms as − 2, − 1, 0, 1, and 2, so there was no need for further processing. The clinical profile has a mixture of continuous and categorical columns describing the demographic and treatment-related details of the patients. It includes age at diagnosis, size, lymph nodes positive, grade, inferred menopausal state, type of breast surgery, type of therapy the patient has gone through (Chemotherapy, Hormone Therapy, and Radio Therapy), and some pathological details of genes and tumors. So, Sun et al. firstly assigned positive numbers for all categorical values and then normalized them using min-max normalization^7^ in the range [0,1]. It brings all the features to the same scale and helps in the training stability of the classifiers. The high dimensionality involved in the genetic data and the low sample size might be the bottleneck in training any deep learning architecture, so the need for feature selection techniques arose. As minimum redundancy maximum relevancy (mRMR)^29^ has the ability to identify the most relevant and informative variables while minimizing redundancy, we applied it to shrank the feature space of gene expression and copy number variation from 24,000 to 400 and 26,000 to 200 features, respectively. The considered features are the most informative genes responsible for breast cancer, which can improve the accuracy of predictive models, enhance our understanding of the disease, and potentially lead to more effective diagnostic and treatment strategies.

## Methodology

**Figure 1:**
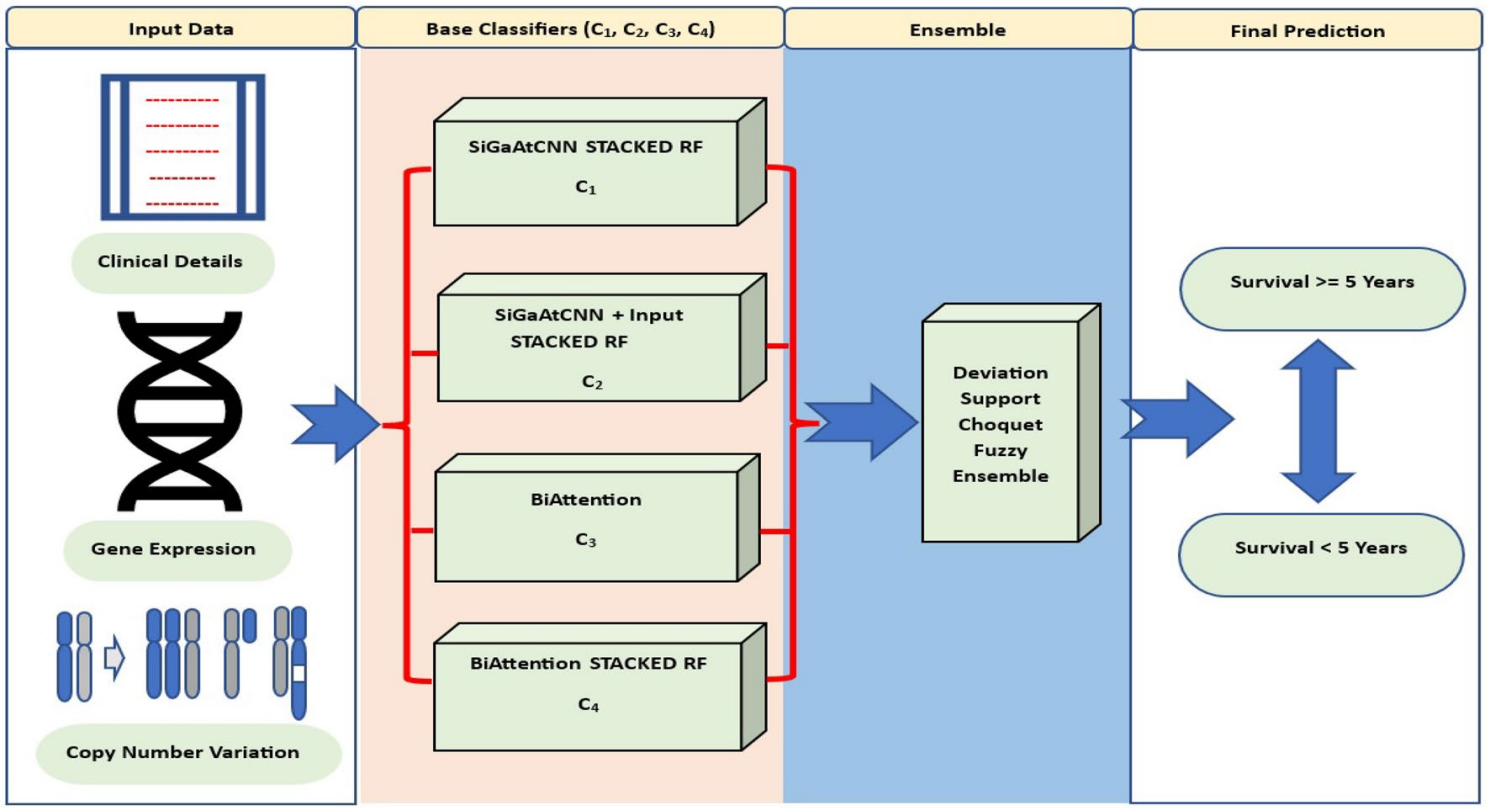
The proposed deviation-support based fuzzy ensemble (*DeSuFEn*) architecture.

This section starts with a brief overview of the state-of-the-art base classifiers proposed by Arya et al.^3^ for the breast cancer survival prediction task. The base classifiers used in this work are SiGaAtCNN STACKED RF^3^, SiGaAtCNN+Input STACKED RF^3^, BiAttention^3^, and BiAttention STACKED RF^3^. These base classifiers are trained on the METABRIC multi-modal breast cancer dataset with the binary classification task of survival prediction. It classifies breast cancer patients into two possible groups (long and short-term survivors) based on the survival cut-off of 5 years. Further, this section details the Deviation-Support based Fuzzy Ensemble (*DeSuFEn*) of prediction probabilities from these base classifiers. The novel ensembling technique utilizes two non-linear functions to get support and deviation terms for each prediction score. Here, the support term acts as a reward, while the deviation term acts as a penalty. The ultimate goal of any classifier is to get the highest prediction score value, 1. If the prediction score is too far from expected, the deviation term shows a higher deviation, and the support term shows less reward. If the prediction score is close to expected, the deviation term signifies a lower deviation, and the support term signifies a higher reward. This proposed approach overcomes the shortcomings of the conventional ensemble methods that do not consider the fact mentioned above^30^, which may lead to an incorrect prediction. We consider the prediction probabilities from different base classifiers over testing data and pass them through two non-linear functions to calculate the deviation and support scores. Further, we integrate these scores using the Choquet fuzzy integral to get the aggregated deviation and support scores corresponding to the expected prediction score. The agreement between the aggregated deviation and support scores toward a particular class provides the final prediction class. In case of a clash where aggregated deviation and support scores do not have a mutual agreement for a particular class, we prioritize the decision supported by the aggregated deviation score. The final proposed architecture is depicted in Fig. 1. The following subsections briefly discuss the architecture of different base classifiers, the mathematical foundation of *DeSuFEn*, and the Choquet fuzzy integral.

### Base classifiers

This section gives a brief architectural overview of four base classifiers used for the ensemble. The first base classifier SiGaAtCNN STACKED RF (C_1_) is a two-stage multi-modal architecture where Arya et al.^3^ have used three different versions of Sigmoid Gated Attention Convolutional Neural Network (SiGaAtCNN) as the feature extractor for three modalities. These CNNs are equipped with the sigmoid gating mechanism, which helps filter out the relevant features from the convolved feature maps of the convolutional layer. In the first stage, the SiGaAtCNNs are trained to extract the informative features and discard the useless and redundant features toward the breast cancer survival prediction goal. The extracted features are integrated and passed to the second stage of the model, where the actual classification task is performed with the help of a random forest classifier. The second base classifier, SiGaAtCNN+Input STACKED RF^3^ (C_2_), is also a two-stage model. It follows a similar architecture as the first base classifier. It differs from the first base classifier in the second stage. The input features passed to the random forest classifier of this model include original input features along with the SiGaAtCNN extracted features to form the stacked features. The authors have integrated these features to handle the scenarios where the CNNs have failed to learn the importance of certain features and have not included them in the extracted features. Injecting the original features into the CNN extracted features serves as complementary information for the classification task. Further, these integrated stacked features are used to perform the final classification task using a random forest classifier.

The third classifier, BiAttention^3^ (C_3_), is a single-stage deep learning architecture. The entire architecture is trained, and its weights are updated in a single pipeline. The architecture uses the SiGaAtCNNs and BiModalAttention as the backbone. The SiGaAtCNNs provide task-oriented max pooled features with the help of the sigmoid gating mechanism. The BiModalAttention applies cross-modality attention to these relevant max pooled features and gets the cross-modality attention features. Further, the cross-modality attentive features obtained in a bi-modal manner from the BiModalAttention layer and the max pooled features from SiGaAtCNNs are integrated and forwarded to dense layers for the final survival classification. The fourth classifier, BiAttention STACKED RF^3^ (C_4_), is a two-stage architecture. Here, the BiAttention architecture (C_3_) is used as the feature extractor and random forest as the classifier. The features extracted from the hidden layers of the BiAttention model are passed to the random forest classifiers for the final classification goal. In the next subsections, we explain how the deviation and support scores are calculated. Moreover, we also provide the mathematical and numerical justification of our proposed fuzzy ensemble technique.

### Deviation-support based fuzzy ensemble (*DeSuFEn*)

This section details the intuition and mathematical foundation of the proposed ensemble technique.

#### Intuition

The deviation-support based fuzzy ensemble combines the outputs of base classifiers. It ensembles by considering the influence of each classifier’s deviation and support value in the final result. The deviation should be reciprocal, while support should be proportional to the prediction score. If the prediction score of a class tends to be 1, the deviation score should tend toward 0. With a gradual decrease in prediction scores, the deviation values should increase. The behavior of the support should be reversed to reward the reliable prediction scores and penalize the lousy prediction scores. We, further apply the Choquet fuzzy integral of deviation and support over all possible base classifiers to get the aggregated deviation and support values.

In the end, we determine the class for each instance based on the mutual agreement between the aggregated deviation and support scores for the predicted class. If there’s no agreement, we prioritize the deviation score. The multiplicative measure of deviation and support validates the intuition of prioritizing deviation over support. In the process of multiplicative measure, the final score is highly influenced by the deviation term. It assigns a lower deviation score for correct predictions. Hence, we are bound to select the class supported by the aggregated deviation score as the final class.

#### Mathematical foundation

While establishing the mathematical foundation of our proposed ensemble, we have used the following notations throughout the study. Here, *N*: Number of classes; *C*: Number of base classifiers; $\mathbb {P}^c_n$: Prediction score of *n*th class by the *c*th base classifier; $D^c_n$: Deviation of the actual prediction score ($\mathbb {P}^c_n$) from the expected prediction score (1); $S^c_n$: Support or Reward score for the prediction score ($\mathbb {P}^c_n$); and $\mathbb {R}_n$: Multiplicative measure of $D^c_n$ and $S^c_n$ for the prediction of *n*th class.

Let $\mathbb {P}^c_n \in \{P^c_1,P^c_2,P^c_3, \ldots ,P^c_N\}$ be the prediction score by classifier *c* for *n*th class, where $c \in \{1,2,3, \ldots ,C\}$ and $n \in \{1,2,3, \ldots ,N\}$. As the prediction scores $\{P^c_1, P^c_2, P^c_3, \ldots , P^c_N\}$ are the probability values, it will follow that summation of all the probabilities for all possible classes equals one. We get the set of prediction scores from all the classifiers and then calculate the deviation and support for each prediction score. Let, $D^c_n \in \{D^c_1, D^c_2, D^c_3, \ldots ,D^c_N\}$ and $S^c_n \in \{S^c_1, S^c_2, S^c_3, \ldots ,S^c_N\}$ be the deviations and support values calculated for $\mathbb {P}^c_n \in \{P^c_1,P^c_2,P^c_3, \ldots ,P^c_N\}$. We define two non-linear functions to generate support and deviation as Eqs. (1) and (2), respectively.

1 $$\begin{aligned} S^{c}_n= & {} 1 - tanh\left\{ \frac{(\mathbb {P}^c_n-1)^2}{2}\right\} \end{aligned}$$

2 $$\begin{aligned} D^{c}_n= & {} 1 - exp\left\{ -\frac{(\mathbb {P}^c_n-1)^2}{2}\right\} \end{aligned}$$

**Figure 2:**
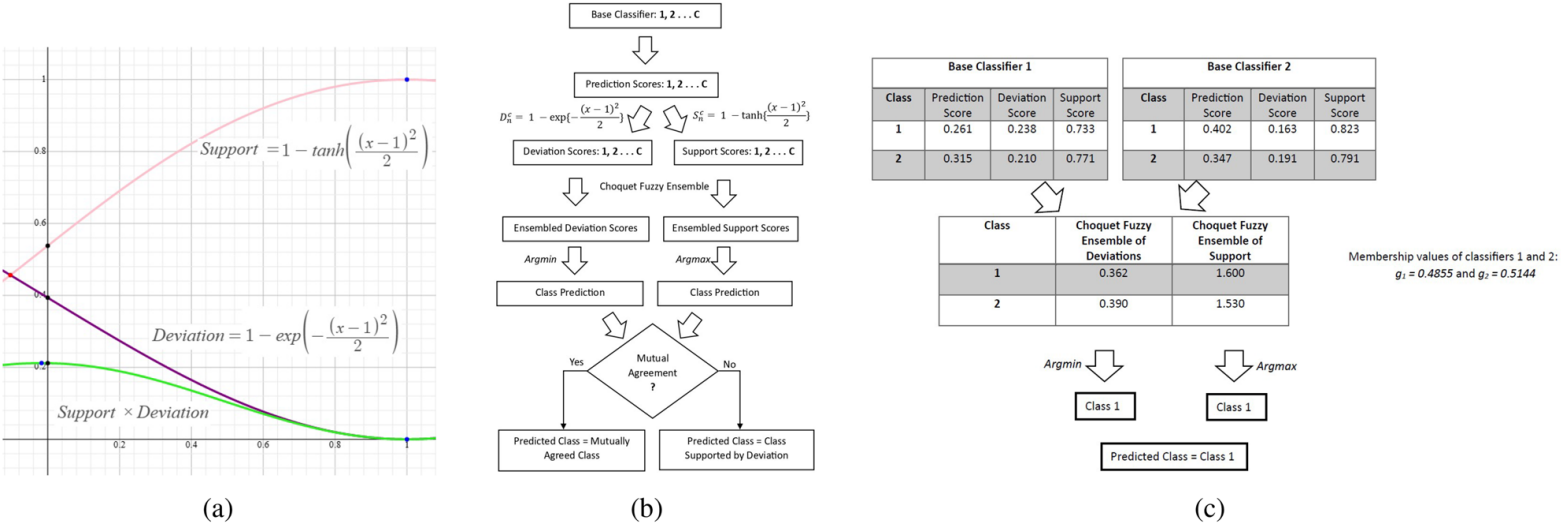
(**a**) Graphical representation of deviation and support functions. Here, horizontal axis : prediction probabilities and vertical axis: deviation and support values (**b**) Flow chart, and (**c**) A numerical example of the proposed method.

As the inputs to these non-linear functions are prediction probabilities, their domain will be $\left[ \left. 0,1\right] \right.$. Function selection is made so their ranges should also be $\left[ \left. 0,1\right] \right.$ for all real numbers. We plotted these two functions in Fig. 2a for better visualization and understanding in the $\left[ \left. 0,1\right] \right.$ domain. From Fig. 2a, these functions support our intuition. If the prediction score is one, the corresponding support is highest, and the deviation is lowest. If we gradually move the prediction score from one to zero, there is a monotonic decrease in the support and an increase in the deviation. Once we obtain the deviation and support scores from base classifiers for each class, these scores need to be aggregated with some ensemble mechanism. For this aggregation, we apply the Choquet fuzzy ensemble as detailed in the following subsection.

### Choquet fuzzy integral

The Choquet fuzzy integral^18, 31^ aims to collect the scores generated by multiple classifiers as a single global score. Here, we will be applying the fuzzy integral over deviation ($D^c_n$) and support ($S^c_n$) scores $\forall \ c\in \{1,2, \ldots , C\}$ to get the global deviation and support scores, represented as $\mathscr {D}_n$ and $\mathscr {S}_n$ $\forall n\in \{1,2, \ldots , N\}$, respectively. The Choquet fuzzy integral-based aggregation technique is selected because it harnesses the degree of uncertainty from the prediction scores and utilizes this as additional information in the fusion of classifiers. The very first step of this aggregation technique is to find the fuzzy measure values (*g*) for the base classifiers and their combinations. The fuzzy measures of base classifier *i* is calculated as $g_i = \frac{acc_i}{acc_1+acc_2+ \cdots +acc_C}$. To get the fuzzy measures for the combination of classifiers, we use Eq. (3).

3 $$\begin{aligned} g_{ij} = g_i+g_j+\lambda g_i g_j \ \forall i, j \in \{1,2, \ldots ,C\} \end{aligned}$$

Here, the fuzzy measure follows two boundary conditions:

- Condition 1: $g_{\phi } =0$ implies that the ensemble strength will be zero if no classifier is involved.
- Condition 2: $g_{12 \ldots C} = 1$ implies that the ensemble strength will be maximum if all the classifiers are involved.

To get the fuzzy measures for the combination of classifiers using Eq. (3), it is required to have the value $\lambda$ which is calculated as the root of Eq. (4), where $-1< \lambda < \infty$.

4 $$\begin{aligned} 1+\lambda = \prod ^C_{i=1}(g_i\lambda +1) \end{aligned}$$

We apply Choquet fuzzy integral using these fuzzy measures over the obtained deviation and support scores as Eq. (5) and 6, respectively, for all possible classes.

5 $$\begin{aligned} \mathscr {D}_n= & {} D^C_n*g_{12 \ldots C}+(D^{C-1}_n - D^C_n)*g_{12 \ldots C-1}+ \cdots +(D^1_n-D^2_n)*g_{1}\nonumber \\{} & {} \quad \forall n\in \{1,2, \ldots ,N\} \end{aligned}$$

6 $$\begin{aligned} \mathscr {S}_n= & {} S^C_n*g_{12 \ldots C}+(S^{C-1}_n - S^C_n)*g_{12...C-1}+ \cdots +(S^1_n-S^2_n)*g_{1}\nonumber \\{} & {} \quad \forall n\in \{1,2, \ldots ,N\} \end{aligned}$$

In the next step, to get the final class label, we apply $argmin(\mathscr {D}_1,\mathscr {D}_2, \ldots ,\mathscr {D}_N)$ and $argmax(\mathscr {S}_1,\mathscr {S}_2, \ldots ,\mathscr {S}_N)$ on the aggregated deviation and support scores over all possible classes, respectively. The minimum deviation value indicates the closeness of the predicted and actual class labels. The maximum support value suggests confidence in the class with a higher prediction probability. If there is a mutual agreement between aggregated deviation and support scores, we select the agreed class as the final prediction. The problem arises when both the terms disagree for a common prediction class. To handle the non-agreement, we prefer the prediction of deviation over support. The rationale behind this decision is as follows: If we consider the multiplicative measure of the support and deviation values calculated as Eq. (7), then the final score for each class is being guided by the deviation term.

7 $$\begin{aligned} \mathbb {R}_n = \sum _{i=1}^{C}{D^i_n \times S^i_n}, \forall n=1,2,3, \ldots ,N \end{aligned}$$

This happens because the range of deviation (Eq. (2)) is less than the range of support (Eq. (1)) in the domain $\left[ \left. 0,1\right] \right.$. Hence, the product of these two terms is dominated by the deviation term (Eq. (2)). It is also evident from the green trajectory in Fig. 2a. The summarized view of end-to-end flow and numerical example for our proposed method is depicted in Fig. 2b and c for better understanding of our proposed method.

## Experimentation and results

This section provides findings of our proposed technique and detailed analyses of all the outcomes.

### Experimental setup

The METABRIC^4^ dataset is small and highly imbalanced, restricting us from performing a simple train-test split-based training. So, we divide them into ten stratified folds and train each of the four base classifiers in a 10-fold cross-validation framework. It ensures that each fold of the dataset contains a representative proportion of the minority class. The model is exposed to the minority class during training and testing in multiple iterations, helping it learn and generalize better for both classes. 10-fold cross-validation reduces the risk of overfitting, ensures a more comprehensive use of the dataset, provides averaged performance metrics, detects variability, and reveals data-dependent errors, all of which contribute to a more robust assessment. The prediction scores of test samples from each fold are generated using all the base classifiers and then ensembled using the proposed *DeSuFEn* to obtain the final class labels. We further observe performance metrics based on the correct and incorrect predictions and take the average of ten folds to report as the final results. The preprocessed data in this study is very lightweight, with small feature space and sample size. There is no need for any extensive computational resources such as GPUs. Hence, all the experiments are performed on the system configured with Windows 11, i7 9th generation processor, 512GB SSD, 4 GB NVIDIA GeForce GTX 1650, and 8 GB RAM. The coding setup has keras 2.2.4 with tensorflow 1.14.0 and Python 3.6 bundled in the Anaconda environment.

### Performance metrics

The proposed ensemble technique is evaluated using some of the traditional performance metrics such as accuracy (*Ac*)^32^, sensitivity (*Sn*)^32^, F_1_-score $({F_{1}-s})$^32^, and balanced accuracy (*Bal-Ac*)^32^ used in the field of machine learning. Here, *Ac* is the ratio of correctly classified instances and total instances, and *Sn* is the ratio of correctly classified positive instances and total predicted positive instances. While ${F_{1}-s}$ and *Bal-Ac* are the mean values of different measures. ${F_{1}-s}$ is calculated as the harmonic mean of precision and sensitivity. *Bal-Ac* is the simple average of sensitivity and specificity. Precision is the ratio of correctly classified positive instances and actual positive instances. Specificity is the ratio of correctly classified negative instances and total predicted negative instances.

### Comparative analyses of base classifiers

The section provides the comparative results of all the four base classifiers^3^, SiGaAtCNN STACKEDRF (C_1_), SiGaAtCNN+Input STACKEDRF (C_2_), BiAttention (C_3_), and BiAttention STACKEDRF (C_4_)) for the breast cancer survival prediction. If we compare the performance metrics of these base classifiers, then observations are as follows:

- C_1_ and C_2_ are the worst performers with ${Ac, Sn, F_{1}-s,}$ and *Bal-Ac* 0.7727, 0.5255, 0.5356, and 0.6899; and 0.7717, 0.5358, 0.5391, and 0.6927, respectively.
- Base classifiers C_3_ and C_4_ dominate the other two worst performers and achieves ${Ac, Sn, F_{1}-s,}$ and *Bal-Ac* of 0.8182, 0.5620, 0.5994, and 0.7323; and 0.8187, 0.5844, 0.6158, and 0.7401, respectively.
- Among best performing base classifiers C_4_ outperforms C_3_ by 0.05%, 2.24%, 1.64%, and 0.78% in terms of *Ac*, *Sn*, ${F_{1}-s}$, and *Bal-Ac*, respectively.

### Comparative analyses of base classifiers and *DeSuFEn*

**Figure 3:**
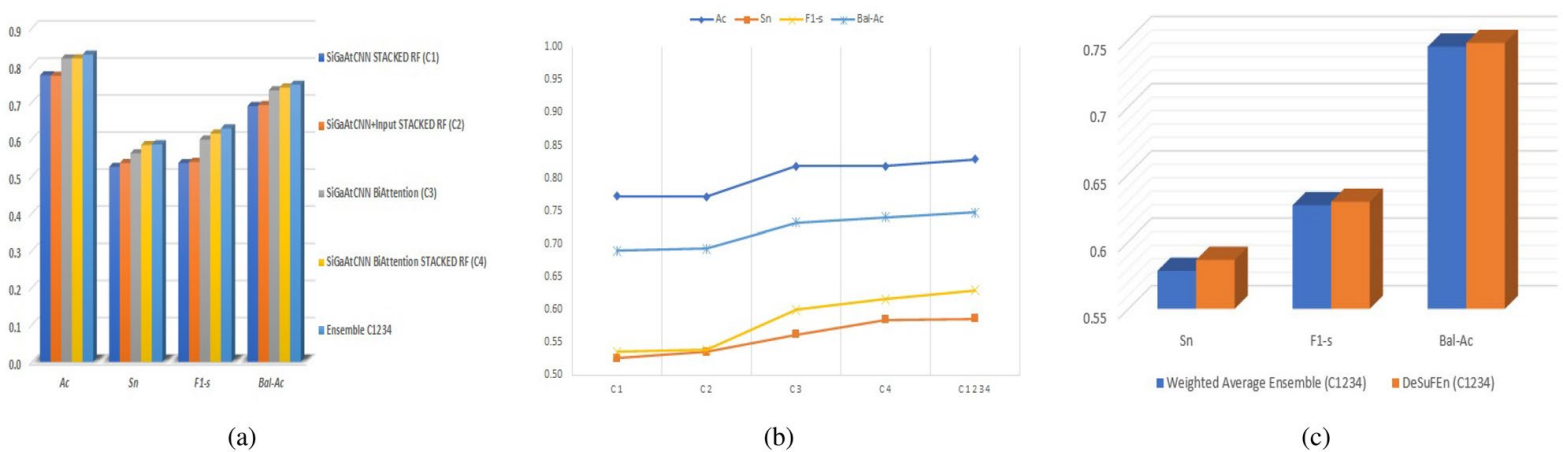
(**a**) Bar graph and (**b**) performance curve for comparative analyses between *DeSuFEn* (C_1234_) and base classifiers. (**c**) Comparative analysis of weighted average ensemble and *DeSuFEn* (C_1234_) methods.

Here, we compare the performance of base classifiers and proposed *DeSuFEn* classifier (C_1234_). From Table 2, we get the ${Ac, Sn, F_{1}-s}$, and *Bal-Ac* of *DeSuFEn* (C_1234_) as 0.8288, 0.5864, 0.6294, and 0.7475, respectively. The obtained performance metrics of our ensemble classifier are 5.61%, 6.09%, 9.38%, and 5.76% higher than the worst-performing base classifier (C_1_) and 1.01%, 0.2%, 1.36%, and 0.74% higher than the best-performing classifier (C_4_). This comparison is depicted in Fig. 3a. We observe here that the ensemble of all four base classifiers outperforms all the base classifiers. Figure 3b shows the inclination in the ${Ac, Sn, F_{1}-s,}$ and *Bal-Ac* values if we move from weaker base classifier to stronger classifiers, toward the ensemble classifier.

**Table 2:** Comparative results of *DeSuFEn* and base classifiers for breast cancer survival estimation.

Models	*Ac*	*Sn*	${F_{1}-s}$	*Bal-Ac*
Weakest base classifier (C_1_)	0.7727	0.5255	0.5356	0.6899
Strongest base classifier (C_4_)	0.8187	0.5844	0.6158	0.7401
*DeSuFEn* (C_1234_)	0.8288	0.5864	0.6294	0.7475

### Comparison with existing techniques

To showcase the superiority of *DeSuFEn* in breast cancer survival prognosis, we perform the comparative analysis with existing state-of-the-art methods from the “Literature Review” section. The results are presented in Table 3. MDNNMD^10^ comes out to be the best model among existing methods and achieves ${Ac, Sn, F_{1}-s,}$ and *Bal-Ac* of 0.7940, 0.2000, 0.3255, and 0.5950, respectively. Our proposed technique overshadow the MDNNMD by 3.48%, 38.64%, 30.39%, and 15.25% in terms of ${Ac, Sn, F_{1}-s,}$ and *Bal-Ac*, respectively. The “weighted average ensemble” is an existing technique used in machine learning. In this approach, multiple base models or classifiers are combined by assigning weights to each model’s predictions. These weights determine how much influence each model has in making the final prediction. The predictions of individual models are multiplied by their respective weights and then summed to produce the ensemble’s prediction. We compare this existing technique with our proposed approach to highlight the differences and advantages of our method. While the weighted average ensemble relies on fixed weights for combining predictions, our method utilizes deviation and support terms to dynamically adjust the influence of each base classifier based on their accuracy and consistency. Table 3 highlights the comparative performance differences of the proposed ensemble and the weighted average ensemble of base classifiers. Our proposed ensemble outperforms the existing popular weighted ensemble in terms of ${Sn, F_{1}-s}$ and *Bal-Ac* as depicted in Fig. 3c.

**Table 3:** Comparative results of *DeSuFEn* and existing methods for breast cancer survival estimation.

Models	*Ac*	*Sn*	${F_{1}-s}$	*Bal-Ac*
MDNNMD^10^	0.7940	0.2000	0.3255	0.5950
SVM^8^	0.7750	0.1220	0.2120	0.5560
RF^9^	0.7700	0.0980	0.1742	0.5440
LR^33^	0.7540	0.0370	0.0694	0.5135
Weighted average Ensemble (C_1234_)	0.8288	0.5783	0.6268	0.7448
*DeSuFEn* (C_1234_)	0.8288	0.5864	0.6294	0.7475

The improvement in the results is the outcome of better feature selection followed by the proposed ensemble mechanism. The more informative and dimension reduced mRMR based features enhanced the generalization ability of the classifiers. The beauty of our ensemble technique lies in the fact that even if we involve the worst performers, the results are highly influenced by the best performers, which is not the case in the traditional ensemble technique. The dynamic adaptability of influence of each base classifier allows our ensemble to potentially outperform traditional weighted average ensembles by providing more accurate and robust predictions, especially in complex and heterogeneous datasets, such as those encountered in breast cancer survival analysis.

## Conclusion

Breast cancer survival analysis holds pivotal importance in healthcare and research for improving patient outcomes, treatment efficacy, and understanding disease progression. Yet, the analysis is beset by formidable challenges due to the heterogeneous nature of breast cancer and the complexity of data sources. The complexity of integrating clinical, imaging, genomic, and pathology data, alongside data quality disparities, further complicates the process. Successfully addressing these challenges requires the judicious use of machine and deep learning, ultimately offering a deeper understanding of breast cancer survival and its potential to enhance diagnostics and treatment strategies. Due to the heterogeneity involved in the data, it becomes difficult for one classifier to learn all possible hidden relationships between multi-modal features and class labels. This forces different classifiers to perform differently for the same set of patients. Hence, we propose the ensemble of state-of-the-art base classifiers to make our prediction more robust. The proposed *DeSuFEn* is a novel and more advanced version of the ensemble technique. In our approach, we enhance the way we combine base classifiers by considering deviation and support terms that account for how well they predict class outcomes. These terms provides the supportive and validation measure of the predicted probabilities, which is missing in traditional ensemble techniques. The Choquet fuzzy integral helps decide the contributions of different basic classifiers, under the influence of deviation and support functions to evaluate how closely their predictions match the expected outcomes. If integrated results of both the functions point to same class then we fix it as predicted class otherwise we rely on the class guided by deviation term. The mathematical foundations and empirical validation aligns together and suggest the superiority of *DeSuFEn* over base classifiers and other existing methods. It outperforms the weakest base classifier by 5.61%, 6.09%, 9.38% and 5.76%, and strongest base classifier by 1.01%, 0.2%, 1.36%, and 0.74% in terms of $Ac, Sn, F_{1}-s$, and *Bal-Ac*, respectively. These results prove that our ensemble can correctly predict instances that the base classifiers got wrong. This highlights the ensemble’s robustness in capturing complex relationships in the data. In the future, we plan to accommodate additional modalities such as whole slide images and DNA methylation in breast cancer survival analysis. This ensemble approach also holds potential for addressing classification challenges in both medical and non-medical fields and adaption of other non-linear functions. The proposed ensemble is a domain independent generic technique which can be effectively applied in various fields and tasks. Our innovative ensemble technique not only significantly improves breast cancer survival prognosis but also holds the promise of transforming the field. With its demonstrated success in enhancing accuracy, sensitivity, and other vital metrics, this approach has the power to make a profound impact on the early detection and treatment of breast cancer, ultimately saving lives.

## Data Availability

The dataset and code used in this study is publicly available at https://www.cbioportal.org/study/summary?id=brca_metabric and https://github.com/nikhilaryan92/DeSuFEn, respectively.
